# Annual incidence of general practice consultations related, according to the general practitioner, to bed bugs and description of cases, 2019–2020, France

**DOI:** 10.1371/journal.pone.0308990

**Published:** 2024-08-27

**Authors:** Bérenger Thomas, Florent Hamaide-Defrocourt, Titouan Launay, Pauline Vasseur, Ophélie Guyonvarch, Patricia Lefébure, Louise Rossignol, Nadia Younès, Clément Turbelin, Caroline Guerrisi, Thomas Hanslik, Thierry Blanchon, Mathieu Rivière, Romain Pons

**Affiliations:** 1 Sorbonne Université, INSERM, Institut Pierre Louis d’Épidémiologie et de Santé Publique, Paris, France; 2 General Practitioner, Limay, France; 3 Département de Médecine Générale, Université Paris Cité, Paris, France; 4 Service de Psychiatrie Adulte, Centre Hospitalier de Versailles, Versailles, France; 5 Team DevPsy, Centre de Recherche en Epidémiologie et Santé des Populations, Université Paris-Saclay, Villejuif, France; 6 Service de Médecine Interne, Hôpital Ambroise Paré, Assistance Publique ‐ Hôpitaux de Paris, Boulogne Billancourt, France; 7 Université de Versailles Saint-Quentin-en-Yvelines, UFR Simone Veil ‐ Santé, Versailles, France; Gomal University, PAKISTAN

## Abstract

Bed bug infestations have been increasing worldwide since the 2000s. Their consequences for health range from skin reactions to major psychological distress. However, epidemiological data is still lacking. This study estimated the incidence of general practice consultations related to bed bugs in France and evaluated factors associated with repercussions on everyday life. We conducted a prospective observational study from March 2019 to April 2020 among 217 GPs in France. Annual and monthly GP consultations related to bed bugs were estimated from the reported cases. Descriptive analyses were performed, and factors associated with repercussions on everyday life were assessed using a logistic regression model. The annual incidence rate of GP consultations related to bed bugs was estimated at 109 per 100,000 inhabitants [95%CI: 92–126]. Bed bug-related consultations occurred in all regions, peaking in the summer. Moderate-to-severe repercussions on everyday life were reported by 39% of the patients. The associated factors with this level of repercussions were: having seen bed bugs (OR = 4.02 [95%CI: 1.6–10.73]), having lesions from scratching (OR = 5.25 [1.65–19.92]), having lesions on the head and the neck (OR = 3.97 [1.52–10.95]) and reporting psychological distress (OR = 6.79 [2.47–20.42]). This study provides new knowledge on GP consultations related to bed bugs in France. These data will help tailor public health programs to the population’s needs, including information and training for primary healthcare professionals.

## Introduction

Bed bugs are hematophagous insects belonging to the Cimicidae that are prevalent worldwide. The development and intensive use of organochlorine and organophosphate insecticides almost eradicated these insects from developed countries during the second half of the 20^th^ century [1]. However, a re-emergence of bed bug infestations associated with the emergence of insecticide resistance and increasing human travel has been observed since the 2000s [2, 3].

Bed bug infestations mainly occur in high-density areas, transit hubs, private homes, and public places such as hotels, public transport, or cinemas [2, 4–6]. Visual inspection can confirm an infestation, but bed bugs are often not correctly identified [7, 8]. Bed bug bites are usually painless [9] and resultant skin lesions, mainly pruritic papules, typically occur in linear or grouped patterns on areas of exposed skin (neck, arms, shoulders and legs) [9–11]. Allergic reactions and anaemia have been reported in a few rare cases. It has also been shown that bed bugs can carry pathogens, but there is currently no evidence of transmission to humans [12, 13]. In addition to cutaneous reactions, bed bug infestations can cause anxiety, stress and insomnia due to itching, fear of insects, social isolation and the cost of eradication measures [14]. According to previous studies, living in shared housing, knowing someone who currently has bed bugs, having a history of infestation, low income, low educational level, young age and the presence of many children and adults in the household are associated with bed bug infestations [15, 16]. Interest of the general population in this public health issue is growing, and studies based on Google Trends reported an increase in the search volume index relating to bed bugs since 2004, with seasonal differences and cyclic peaks in the summer [17, 18].

With the spread of bed bug infestations, general practitioners (GPs) will likely face this issue increasingly in primary care. In France, GPs are the first healthcare professionals to be contacted in the event of clinical repercussions, which are common in case of bed bug infestation. However, to our knowledge, no study providing reliable data about consultations related to bed bugs has been conducted in general practice. The aim of this study was to estimate the incidence of primary care consultations related to bed bugs in France according to the GP, and to describe infestations and clinical characteristics of cases, as well as assessing the repercussions on everyday life for these patients. A secondary objective was to measure GPs’ perception about their own knowledge of bed bugs.

## Materials and methods

### Study design and data collection

We conducted a prospective observational study among GPs from the French *Sentinelles* network, a real-time epidemiologic surveillance system including about 1,300 volunteer GPs (2% of the total number of GPs in France) [19]. The representativeness of *Sentinelles* GPs has been studied before, and some limited differences with all French GPs were noted concerning practice location, sex and number of consultations per week [22]. Expecting 1 to 2 cases per GP per year to ensure that the incidence of consultations could be estimated with a 95% confidence interval equal to +/- 10% around point estimates, we recruited 217 GPs for this study (initial aim: 215). The regional recruitment of participating GPs followed the regional distribution of French GPs (S1 Table). GP feelings about their knowledge of bed bugs was assessed at the beginning of the study. This included their ability to identify bed bugs, their knowledge of control measures, and their knowledge about symptoms and clinical signs related to bed bugs. Each kind of knowledge was self-rated on a scale from zero (no knowledge) to 10 (complete knowledge).

From March 2019 to April 2020, these GPs were asked to report consultations related, according to them, to bed bugs. Eligible cases were defined as patients seen in general practice consultations presenting symptoms and/or clinical signs related to bed bugs. GPs completed a questionnaire for each patient, providing sociodemographic characteristics (age, sex, occupational status), symptoms and clinical signs (skin lesions, insomnia, psychological distress in the last four weeks (evaluated by Mental Health Inventory test [20]), and degradation of psychological health caused by bed bugs), repercussions on everyday life in three major domains (work, social life/leisure activities, and family life/home responsibilities) over the last 14 days (assessed with the Sheehan Disability Scale, which runs from 0 (no repercussions) to 10 (extreme repercussions)), a description of the infestation (infestation’s place, bed bugs seen), the type of housing, the control measures used, and the treatment prescribed.

### Data management

GP feelings about their knowledge of bed bugs were classified into three categories: “poor” (0 to 3 out of 10), “moderate” (4 to 6) and “good” (7 to 10). The five-question Mental Health Inventory assessed the patient’s psychological distress in the last four weeks [20]. It was considered to be positive (meaning “a good bit of the time”, “most of the time”, or “all of the time”) if the patient felt frequently “nervous” and/or “downhearted and blue” and/or “so down in the dumps that nothing could cheer them up”. Repercussions on everyday life for each domain were considered moderate-to-severe if a score of 4 or higher was obtained [21]. Global repercussions were rated as moderate-to-severe if at least one of the three domains was affected by moderate-to-severe repercussions.

### Statistical analysis

The annual incidence rate of general practice consultations related to bed bugs in France was estimated as the mean number of cases notified by the *Sentinelles* GPs (adjusted for participation and geographic distribution) multiplied by the total number of GPs practicing in France and divided by population size (census data) [22]. We estimated annual incidence rates by age group and sex and monthly incidence rates. The variation of incidence over the four quarters of the year (April to June, July to September, October to December 2019 and January to March 2020) was assessed with a Kruskal-Wallis rank sum test followed by a Dunn’s post hoc test.

We performed a descriptive analysis of the participating GPs and reported cases. We used Pearson’s chi-squared test or Fisher’s exact test (categorical variables) and Kruskal-Wallis rank sum tests (continuous variables) to evaluate the significance of differences. Using a univariate and multivariate logistic regression model, we investigated the factors associated with moderate-to-severe repercussions on everyday life. A stepwise backward elimination process performed the multivariate analysis until the Akaike information criterion reached a minimum. All analyses were conducted in R software version 3.5.0 [23].

### Ethical considerations

This study was approved by the French Protection of Individuals Committee (CPP). It was authorized by the French National Data Protection Agency (CNIL) as part of ad hoc studies conducted by the Sentinelles network (notice 471 393). Patients were informed by the participating GPs, and oral consent was obtained and witnessed by GPs. In the case of minors, consent was obtained from parents or guardians.

## Results

### Characteristics of the participating GPs

Among the 217 included GPs, 214 performed at least one declaration during the study period (participation rate: 98.6%). More than half (*n* = 109) reported at least one consultation related to bed bugs. The mean number of consultations reported per GP was 1.8 (standard deviation: 1.2; maximum: 10).

The 214 participating GPs had a mean age of 51 (Table 1). Almost two-thirds were male (*n* = 135, 63%), and most worked in an urban area (*n* = 175, 82%). GPs’ feelings about their knowledge of bed bugs revealed that 38% (*n* = 81) considered their ability to identify bed bugs, 17% (*n* = 36) “good” their knowledge about control measure, and 53% (*n* = 112) as “good” their knowledge about the type of symptoms and clinical signs related to bed bugs. GP confidence in their knowledge regarding symptoms and clinical signs related to bed bugs was lower among GPs reporting no cases during the study period (*p =* 0.02).

**Table 1:** Sociodemographic characteristics of participating GPs and GPs’ feelings about their knowledge of bed bugs, according to whether they reported a consultation related to bed bugs during the study*.

	All GPs, n = 214	GPs who reported at least one-bed bugs case, n = 109	GPs who reported no bed bugs case, n = 105	
	no. (%) or mean (SD)	no. (%) or mean (SD)	no. (%) or mean (SD)	p-value**
Mean age, y (SD)	50.94 (12.19)	51.25 (12.34)	50.61 (12.07)	0.71
Sex				0.29
Female	79 (37)	36 (33)	43 (41)	
Male	135 (63)	73 (67)	62 (59)	
Practice type				0.57
Individual	65 (33)	36 (35)	29 (31)	
Group of 2 or 3	85 (43)	41 (39)	44 (47)	
Group of 4 or more	48 (24)	27 (26)	21 (22)	
Age at establishment time, y				0.34
< 30	67 (32)	39 (36)	28 (27)	
30–34	110 (52)	56 (51)	54 (53)	
35–39	24 (11)	11 (10)	13 (13)	
≥ 40	10 (5)	3 (3)	7 (7)	
Area of practice				0.63
Urban	175 (82)	91 (83)	84 (80)	
Rural	39 (18)	18 (17)	21 (20)	
Self-evaluation of knowledge				
Ability to identify bed bugs				0.22
Poor	62 (29)	26 (24)	36 (35)	
Moderate	70 (33)	38 (35)	32 (31)	
Good	81 (38)	45 (41)	36 (35)	
Knowledge about control techniques				0.45
Poor	73 (34)	33 (30)	40 (38)	
Moderate	104 (49)	56 (51)	48 (46)	
Good	36 (17)	20 (18)	16 (15)	
Knowledge about symptoms and clinical signs				0.02
Poor	34 (16)	10 (9)	24 (23)	
Moderate	67 (31)	39 (36)	28 (27)	
Good	112 (53)	60 (55)	52 (50)	

*Data were missing for the following characteristics: age (3), practice type (16), age at establishment time (3), self-evaluation of knowledge (1). GP, general practitioner; SD: standard deviation

**Categorical variables: Pearson’s chi-squared test or Fisher’s exact test; continuous variables: Kruskal-Wallis rank sum tests

### Incidence of GP consultations related to bed bugs

The annual incidence of GP consultations related, according to them, to bed bugs was estimated at 71,925 [95% confidence interval: 60,606–83,244] between April 2019 and March 2020, corresponding to an annual incidence rate of 109 consultations per 100,000 inhabitants [95%CI: 92; 126] (Table 2). The annual incidence rates by sex were estimated at 139 [95%CI: 112; 166] per 100,000 women and 80 [95%CI: 59; 101] per 100,000 men. Based on age, the two categories with the highest annual incidence rates were individuals under 20 and those aged 20 to 29. Cases were reported in all regions of mainland France, with annual incidence rates ranging from 19 [95%CI: 0; 46] to 216 [95%CI: 53; 329] consultations per 100,000 inhabitants.

**Table 2:** National annual incidence and incidence rates of GPs’ consultations related to bed bugs in France between April 2019 and March 2020 according to GP reports (global incidence and incidences by gender and age).

	Incidence [CI95%]	Incidence rate [CI95%]
		(GPs’ consultations per 100,000 inhabitants)
Total	71,925 [60,606–83,244]	109 [92–126]
By sex		
Female	46,561 [37,432–55,690]	139 [112–166]
Male	25,109 [18,437 – 31,782]	80 [59–101]
By age, y		
0–19	20,452 [14,426 – 26,477]	130 [92–169]
20–29	14,493 [9,312 – 19,675]	196 [126–265]
30–49	14,194 [9,254 – 19,134]	86 [56–116]
≥ 50	22,531 [16,182 – 28,880]	89 [64–115]

**Fig 1:**
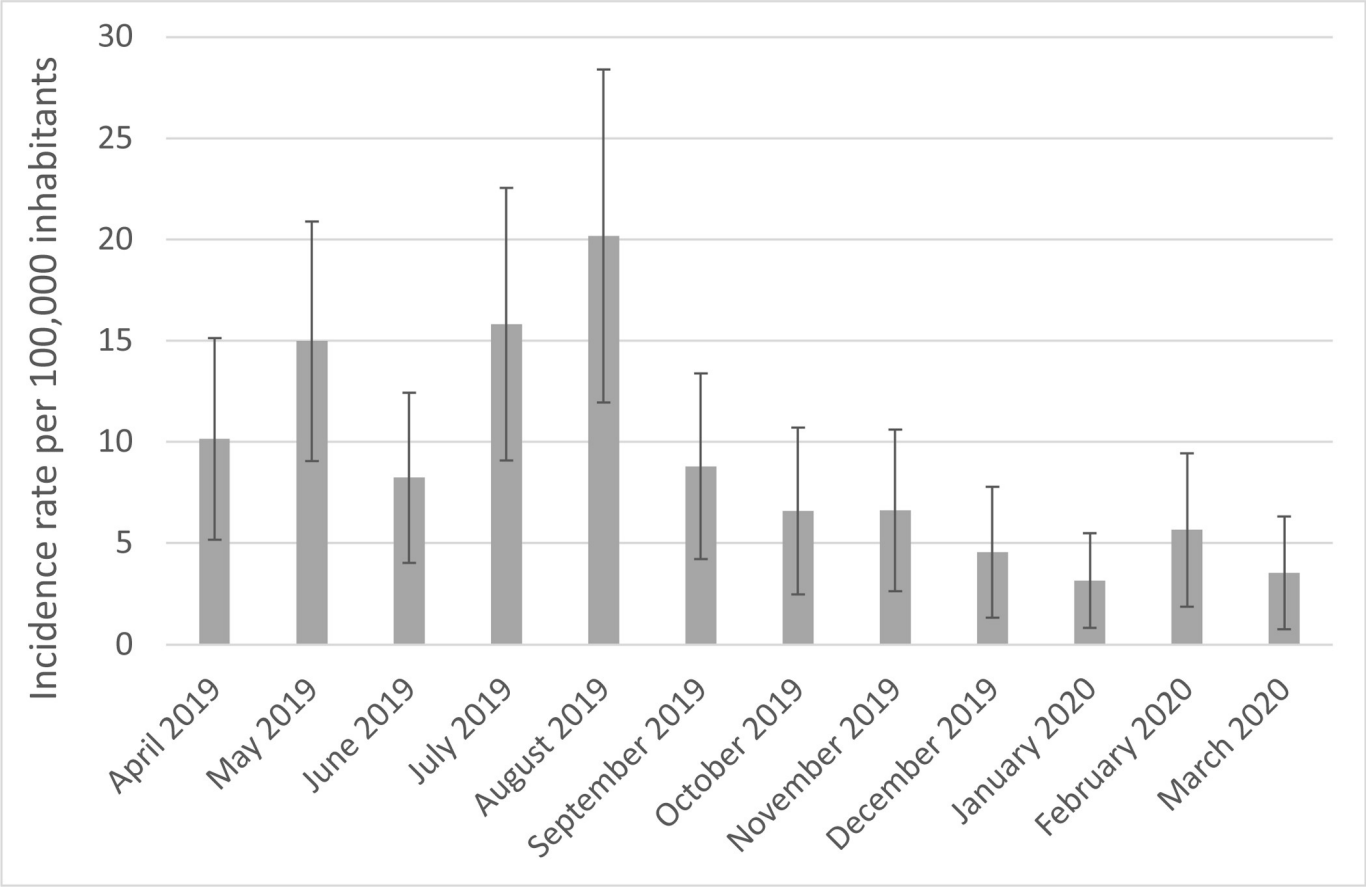
Estimated monthly incidence rates per 100,000 inhabitants (and 95% confidence intervals) of general practice consultations related to bed bugs in France between April 2019 and March 2020 according to GP reports.

Monthly annual incidence rates ranged from 3 [95%CI: 1; 5] consultations per 100,000 inhabitants in January 2020 to 20 [95%CI: 12; 28] in August 2019 (Fig 1). Incidence rates were significantly higher in spring and summer 2019 (the first quarters April-June 2019 and July-September 2019) than in the following winter (January-March 2020) (*p* = 0.02 and 0.003, respectively).

### Description of cases

During the study period, the GPs reported 193 consultations on bed bugs, 191 of which were described (99%). The average age of patients was 34 years, ranging from 8 months to 86 years, and two-thirds were women (*n* = 127, 66%) (Table 3). In more than half of all cases, the bed bug infestation occurred in the patient’s home (*n* = 96, 53%). Bed bugs were seen by the patient, the GP or another person in 48% of cases (*n* = 85). The patient had seen bed bugs in 34% of cases (*n* = 60) and GPs only in 7% (*n* = 13). Control measures were applied in almost two-thirds of home infestations, shared accommodation excluded (*n* = 59, 63%) (S2 Table), mainly through the treatment of laundry (*n* = 49, 83%), through household treatment (vacuuming, steaming, etc) (*n* = 41, 69%) and insecticide use (*n* = 30, 51%). Professional extermination was sought in less than 10% of the cases (*n* = 8, 9%).

**Table 3:** Sociodemographic characteristics of patients seen in general practice consultations related to bed bugs in France between March 2019 and April 2020 and infestation features according to GP reports*.

	n = 191	
	no. or mean	% or SD
Age, y		
Mean (SD)	34.21	21.45
0–19	58	30
20–29	35	18
30–49	43	23
≥ 50	55	29
Sex		
Female	127	66
Male	64	34
Occupational status		
Student	52	28
Working	76	42
Not-working	33	18
Retired	22	12
Infestation location		
Home	96	53
Other private accommodation (family, friend…)	30	16
Shared accommodation (hotels, holiday rentals…)	46	25
Other (transport, workplace…)	10	5
Bed bugs seen by the patient, the GP or another person	85	48
Bed bugs seen by the patient	60	34
Bed bugs seen by the general practitioner	13	7
Another person sees bed bugs	27	15

*Data were missing for the following characteristics: occupational status (8), infestation’s place (9), bed bugs seen (15). GP, general practitioner; SD, standard deviation

### Consultations and clinical descriptions

Half of the consultations (*n* = 95, 51%) occurred within a week of the symptom onset (Table 4). Clinical examination revealed skin lesions in all but three of the patients (*n* = 188, 98%). These lesions mainly were papules (*n* = 155, 82%) and lesions from scratching (*n* = 126, 67%). The upper limbs and the trunk were the most affected area (*n* = 135, 75% and *n* = 125, 69%, respectively). Insomnia was reported by 73 patients (39%), more than a quarter of the patients reported a feeling of psychological distress in the last four weeks (*n* = 40, 27%). Medication was prescribed in most cases (*n* = 172, 91%). Antihistamines were the drugs most frequently prescribed (*n* = 145, 84%).

**Table 4:** Consultations characteristics, clinical description and medical care among patients seen in general practice consultations relating to bed bugs in France between March 2019 and April 2020*.

	n = 191	
	No.	%
History of previous bed bugs symptoms	12	7
Consultation with another health professional for the ongoing symptoms	28	15
of which general practitioner	12	43
of which dermatologist	2	7
of which pharmacist	8	29
of which emergency service	4	14
Time elapsed between symptom onset and consultation		
Less than a week	95	51
Between a week and a month	69	37
More than a month	24	13
Area of practice of consulted general practitioner		
Urban	162	85
Rural	29	15
Bed bugs as the principal reason given for consultation	151	79
Skin lesions	188	98
of which were papules	155	82
of which were lesions from scratching	126	67
of which were macules	73	39
of which were nodules	43	23
of which were ulcers	19	10
of which were superinfections	12	6
of which were blisters	9	5
In the case of skin lesions, affected areas		
Head or neck	58	32
Trunk	125	69
Upper limbs	135	75
Lower limbs	98	66
Insomnia	73	39
Psychological distress in the last four weeks	40	27
Degradation of psychological health caused by bed bugs	26	15
Moderate-to-severe repercussions in the last 14 days		
On work/studies	24	21
On social life/leisure activities	34	22
On family life/home responsibilities	43	28
Globally, in everyday life	60	39
Medications prescribed at the end of the consultation	172	91
of which were antihistamines	145	84
of which were antiseptics	68	40
of which were topical corticosteroids	65	38
of which were topical antibiotics	4	2
of which were oral antibiotics	3	2
of which were anxiolytics	3	2
of which were hypnotics	1	1
of which were antidepressants	0	0
Sick leave certificate	4	2

*Data were missing for the following characteristics: history of previous bed bugs symptoms (7), consultation of another health professional (2), time elapsed between symptom onset and consultation (3), affected areas (8), insomnia (6), psychological distress (43), degradation of psychological health (17), repercussions on work/studies (19), on social life (37), on family life (37), on everyday life (36), medications (2), sick leave certificate (8).

### Factors associated with repercussions on everyday life

More than a third of patients reported moderate-to-severe repercussions on their everyday life in the last 14 days (*n* = 60, 39%) (Table 4). In multivariate analysis (Table 5), factors positively associated with these repercussions were: having seen bed bugs (odds ratio (OR) = 4.02 [95%CI: 1.6–10.73]), having lesions from scratching (OR = 5.25 [1.65–19.92]), having lesions on the head or the neck (OR = 3.97 [1.52–10.95]) and reporting psychological distress in the last four weeks (OR = 6.79 [2.47–20.42]).

**Table 5:** Factors associated with moderate-to-severe repercussions on everyday life in the last 14 days among patients seen in general practice consultations relating to bed bugs in France between March 2019 and April 2020 (univariate and multivariate analyses)*.

			Moderate-to-severe repercussions on everyday life	Univariate analysis		Multivariate analysis	
		No.	no. (%)	OR [95%CI]	p-value	OR [95%CI]	p-value
Sex	Female	103	39 (38)	Ref.	0.76		
	Male	52	21 (40)	1.11 [0.56–2.19]			
Age, y	0–19	39	11 (28)	Ref.	0.40		
	20–29	27	11 (41)	1.75 [0.62–5]			
	30–49	41	16 (39)	1.63 [0.64–4.24]			
	≥ 50	48	22 (46)	2.15 [0.89–5.43]			
Occupational status	Working	71	29 (41)	Ref.	0.38		
	Student	42	12 (29)	0.58 [0.25–1.3]			
	Non-working	24	10 (42)	1.03 [0.4–2.63]			
	Retired	18	9 (50)	1.45 [0.51–4.14]			
Infestation location	Home	76	34 (45)	Ref.	0.15		
	Other private acc.	22	5 (23)	0.36 [0.11–1.03]			
	Shared acc.	42	14 (33)	0.62 [0.28–1.34]			
	Other	9	5 (56)	1.54 [0.38–6.66]			
Bed bugs seen	No	72	18 (25)	Ref.	0.003	Ref.	0.003
	Yes	72	35 (49)	2.84 [1.42–5.84]		4.02 [1.6–10.73]	
History of bed bugs	No	142	53 (37)	Ref.	0.28		
	Yes	9	5 (56)	2.1 [0.53–8.81]			
Cs. with another HP	No	134	50 (37)	Ref.	0.40		
	Yes	19	9 (47)	1.51 [0.56–4]			
Time between symptom onset and cs.	< 1 wk	78	27 (35)	Ref.	0.43		
	1 wk to 1 mo	53	20 (38)	1.14 [0.55–2.36]			
	> 1 mo	22	11 (50)	1.89 [0.72–4.97]			
Practice area	Urban	133	55 (41)	Ref.	0.09		
	Rural	22	5 (23)	0.42 [0.13–1.13]			
Bed bugs are the principal reason given for cs.	No	32	10 (31)	Ref.	0.33		
	Yes	132	50 (41)	1.51 [0.67–3.58]			
Papules	No	29	16 (55)	Ref.	0.05		
	Yes	123	43 (35)	0.44 [0.19–0.99]			
Lesions from scratching	No	42	11 (26)	Ref.	0.04	Ref.	0.004
	Yes	110	48 (44)	2.18 [1.02–4.95]		5.25 [1.65–19.92]	
Macules	No	95	38 (40)	Ref.	0.70		
	Yes	57	21 (37)	0.88 [0.44–1.72]			
Nodules	No	114	44 (39)	Ref.	0.92		
	Yes	38	15 (39)	1.04 [0.48–2.19]			
Ulcers	No	16	7 (44)	Ref.	0.67		
	Yes	136	52 (38)	1.26 [0.43–3.57]			
Superinfection	No	10	5 (50)	Ref.	0.46		
	Yes	142	54 (38)	1.63 [0.43–6.11]			
Blisters	No	7	3 (43)	Ref.	0.82		
	Yes	145	56 (39)	1.19 [0.23–5.6]			
Head and/or neck lesions	No	102	32 (31)	Ref.	0.003	Ref.	0.005
	Yes	43	25 (58)	3.04 [1.47–6.43]		3.97 [1.52–10.95]	
Psychological distress	No	101	28 (28)	Ref.	< 10^−4^	Ref.	< 10^−3^
	Yes	35	24 (69)	5.69 [2.52–13.56]		6.79 [2.47–20.42]	

*acc., accommodation; CI, confidence interval; cs., consultation; HP, health professional; OR, odds ratio

## Discussion

This study provides new knowledge on GP consultations on bed bugs in France according to GP reports. The data includes an estimate of the incidence of consultations, a description of the characteristics of cases, and an assessment of repercussions on patient everyday life.

This is the first study assessing the incidence of general practice consultations related to bed bugs in a country. Most of the published data to date concern reports from pest management companies [2, 4, 15, 16, 24], self-reported declarations or limited local surveys reporting the prevalence of infected households in some cities [25, 26]. Other studies have focused on descriptions of dermatological consultations [27] or consultations at emergency departments [28]. These data complement those regarding the number of interventions from the French national pest management companies reported in 2019 (540,000 interventions) [29] and those of a US emergency department which published an estimate that 27% of patients suffering from bed bugs had consulted a primary care physician [30]. Regarding seasonality, the number of consultations was higher during the spring and summer months, consistent with previous findings. Data from the Danish Pest Infestation Laboratory and Norwegian pest control operators have shown that the number of cases reported is higher from July to November [31]. More extensive studies based on internet search analyses also found seasonality, with peaks in summer in the United States, Europe, and Australia [17, 18].

The incidence rate of GP consultations related to bed bugs was higher among women and children in our study. This result corroborates previous studies [26]. This does not necessarily mean that women or children are more likely to be affected by bed bugs. The incidence rate may reflect different behaviours in seeking healthcare, with women or children turning to their GPs more quickly and often than men, as shown in prior research [32, 33]. Some authors suggested, without any confirmation, that women and children may have a high sensitivity to bed bugs due to more delicate skin [27]. However, a US study assessing reactions to bed bugs showed no significant difference in sensitivity to bites between men and women [11].

Symptoms and clinical signs reported in this study by patients were similar to those previously reported [10]. As often reported in clinical studies, lesions were mainly located on exposed body parts [11, 27, 34]. Their frequencies are not precisely documented. In a US study on 474 patients, skin reactions were reported by 70% of respondents, 72% of whom had itchy red welts [11]. Studies and case reports also often mention psychological consequences [14]. In our study, insomnia and psychological distress were observed in 39% and 27% of patients, respectively. These findings are similar to those of a previous study on customers of US pest control services, in which 29% of patients reported insomnia, 22% mentioned “emotional distress”, 20% had anxiety and 14% experienced stress [11]. Only a few studies have used evaluation scores [35, 36]. Our results, based on validated mental health scales, support previous findings and highlight the significant effect of infestations on mental health.

The main limitation of this study is the absence, for each case, of confirmation of bed bug infestation by an entomologist or disinfestation professional. The cases reported in this study were linked to bed bugs by GPs following information they received from their patients. The incidence rate may be overestimated because GPs could have wrongly linked some consultations to bed bugs. However, this study reflects actual life practices, where GPs cannot investigate bed bug infestation and must rely on questioning to make a diagnosis. Considering this limitation, this study has made it possible to establish an imperfect but essential estimate of the burden of disease in general practice. Another limitation is that incidences were based on declarative data reported by volunteer GPs. Participant GPs may have forgotten some cases. Alternatively, GPs may not have participated because they never saw bed bug infestations among their patients. GP participation was monitored during the study, mitigating incorrect reporting of bed bugs. In the absence of active participation, a reminder was sent by e-mail, and the GPs were contacted by telephone if they remained inactive. Finally, we collected only few data specifically on the infestation. This information was difficult for GPs to collect and investigate during consultations. To improve their participation to the study, we decided to limit the size of the questionnaire and to focus on the main objective of our study dealing with the clinical repercussion of bed bugs.

Despite these limits, this study estimated the burden of disease in the general practice of bed bug infestations and their clinical and psychological repercussions for patients. These data highlight the need for training for primary healthcare professionals. Improving their knowledge and capacity to confirm the link between symptoms and bed bug infestations could enhance patient care and orientation. These results will help tailor public health programs to the population’s specific needs.

## Supporting information

S1 Table Regional repartition of the French population (census data) and of French general practitioners (GPs), target number of recruited GPs for the study and final repartition of recruited GPs. (DOCX)

S2 Table Home characteristics and use of control measures in case of home infestations reported by patients seen in general practice consultations related to bed bugs in France between March 2019 and April 2020*. (DOCX)

## Abbreviations

OR: odds ratio
CI: confidence interval
GP: general practitioner
