# Supplementary material for: Annual incidence of general practice consultations related, according to the general practitioner, to bed bugs and description of cases, 2019–2020, France
Source: PLoS One. 2024 Aug 27;19(8):e0308990. doi: 10.1371/journal.pone.0308990 (PMC11349226; doi:10.1371/journal.pone.0308990)
Supplement: S1 Table — (DOCX) [file pone.0308990.s001.docx]

**S1 Table.** Regional repartition of the French population (census data) and of French general practitioners (GPs), target number of recruited GPs for the study and final repartition of recruited GPs

|  | French population | French GPs | Target number of GPs | Recruited GPs |
| --- | --- | --- | --- | --- |
| Region |  |  |  |  |
| Auvergne-Rhône-Alpes | 7,956,770 | 7,596 | 27 | 27 |
| Bourgogne-Franche-Comté | 2,907,114 | 2,543 | 9 | 11 |
| Bretagne | 3,361,496 | 3,132 | 11 | 13 |
| Centre-Val de Loire | 2,641,391 | 2,126 | 7 | 9 |
| Corse | 325,510 | 300 | 1 | 5 |
| Grand Est | 5,679,877 | 5,291 | 18 | 23 |
| Hauts-de-France | 6,101,843 | 5,577 | 19 | 21 |
| Île-de-France | 12,116,367 | 9,792 | 34 | 31 |
| Normandie | 3,416,175 | 3,011 | 11 | 10 |
| Nouvelle-Aquitaine | 6,010,982 | 6,409 | 22 | 17 |
| Occitanie | 5,827,627 | 6,462 | 23 | 25 |
| Pays de la Loire | 3,765,802 | 3,268 | 11 | 10 |
| Provence-Alpes-Côte d’Azur | 5,039,311 | 6,028 | 21 | 15 |
| Total | 65,150,265 | 61,535 | 215 | 217 |
