# Supplementary material for: Annual incidence of general practice consultations related, according to the general practitioner, to bed bugs and description of cases, 2019–2020, France
Source: PLoS One. 2024 Aug 27;19(8):e0308990. doi: 10.1371/journal.pone.0308990 (PMC11349226; doi:10.1371/journal.pone.0308990)
Supplement: S2 Table — (DOCX) [file pone.0308990.s002.docx]

**S2 Table.** Home characteristics and use of control measures in case of home infestations reported by patients seen in general practice consultations related to bed bugs in France between March 2019 and April 2020 *

|  | n = 96 | |
| --- | --- | --- |
|  | no. | % |
| Housing |  |  |
| Apartment | 55 | 59 |
| House | 31 | 33 |
| Shared accommodation | 7 | 8 |
| Among home infestations except shared accommodation | | |
| Housing owner | 24 | 29 |
| Surface per inhabitant in the housing, m² |  |  |
| 0-19 | 14 | 22 |
| 20-29 | 15 | 23 |
| 30-39 | 12 | 18 |
| ≥ 40 | 24 | 37 |
| Alone in the housing | 20 | 24 |
| Use of control measures | 59 | 63 |
| of which laundry treatment | 49 | 83 |
| of which housing treatment | 41 | 69 |
| of which use of insecticides | 30 | 51 |
| of which removal of laundry or furniture | 23 | 39 |
| of which intervention of a disinfestation professional | 8 | 9 |
| of which use of traps | 5 | 8 |
| Control measures perceived as effective (total or partial eradication) | 18 | 46 |
| Moderate to marked impact on household budget | 12 | 24 |

*Data were missing for the following characteristics: housing (3), housing owner (7), surface per inhabitant (24), alone in the housing (4), use of control measures (2), intervention of a disinfestation professional (2), control measures perceived as effective (20), impact on household budget (9)
